# CD147 induces asthmatic airway remodeling and activation of circulating fibrocytes in a mouse model of asthma

**DOI:** 10.1186/s12931-023-02646-5

**Published:** 2024-01-04

**Authors:** Zhao Li, Tao Cheng, Yaning Guo, Rong Gao, Xuankun Ma, Xuecong Mao, Xinpeng Han

**Affiliations:** Department of Cardiopulmonary Diseases, Xi ’an International Medical Center Hospital, No. 777 of Xitai Road, High-tech Zone, Xi ’an, Shaanxi Province 710100 China

**Keywords:** Asthma, CD147, Airway remodeling, Circulating fibrocytes

## Abstract

**Background:**

Airway remodeling is a poorly reversible feature of asthma which lacks effective therapeutic interventions. CD147 can regulate extracellular matrix (ECM) remodeling and tissue fibrosis, and participate in the pathogenesis of asthma. In this study, the role of CD147 in airway remodeling and activation of circulating fibrocytes was investigated in asthmatic mice.

**Methods:**

Asthmatic mouse model was established by sensitizing and challenging mice with ovalbumin (OVA), and treated with anti-CD147 or Isotype antibody. The number of eosinophils in bronchoalveolar lavage fluid (BALF) was examined by microscope, and the levels of interleukin-4 (IL-4), IL-5 and IL-13 in BALF were detected by enzyme-linked immunosorbent assay (ELISA). The number of CD45^+^ and collagen I (COL-I)^+^ circulating fibrocytes in BALF was detected by flow cytometry. Lung tissue sections were respectively stained with hematoxylin and eosin (HE), periodic acid-Schiff (PAS) or Masson trichrome staining, or used for immunohistochemistry of CD31 and immunohistofluorescence of α-smooth muscle actin (α-SMA), CD45 and COL-I. The protein expression of α-SMA, vascular endothelial growth factor (VEGF), transforming growth factor-β1 (TGF-β1), Fibronectin, and COL-I was determined by western blotting.

**Results:**

Anti-CD147 treatment significantly reduced the number of eosinophils and the levels of IL-4, IL-13, and IL-5 in BALF, and repressed airway inflammatory infiltration and airway wall thickening in asthmatic mice. Anti-CD147 treatment also reduced airway goblet cell metaplasia, collagen deposition, and angiogenesis in asthmatic mice, accompanied by inhibition of VEGF and α-SMA expression. The number of CD45^+^COL-I^+^ circulating fibrocytes was increased in BALF and lung tissues of OVA-induced asthmatic mice, but was decreased by anti-CD147 treatment. In addition, anti-CD147 treatment also reduced the protein expression of COL-I, fibronectin, and TGF-β1 in lung tissues of asthmatic mice.

**Conclusion:**

OVA-triggered airway inflammation and airway remodeling in asthmatic mice can be repressed by anti-CD147 treatment, along with inhibiting the accumulation and activation of circulating fibrocytes.

**Supplementary Information:**

The online version contains supplementary material available at 10.1186/s12931-023-02646-5.

## Introduction

Asthma, usually manifested with airway inflammation and airway hyperresponsiveness [1], is a common and frequent respiratory disease that affects nearly 300 million people all around the world [2]. Although current therapies for asthma, such as corticosteroids and bronchodilators, are effective in suppressing airway inflammation, relieving asthma symptoms, and reducing the risk of death in most asthmatic individuals [3], however, there are still some patients respond poorly to existing therapies and develop adverse outcomes. Moreover, current therapies exhibit only little effect on poorly reversible respiratory pathological changes, such as airway stenosis caused by airway remodeling [4]. Airway remodeling is a key poorly reversible characteristic of asthma, which refers to the chronic structural changes of airway tissues in patients with asthma [5]. These changes including bronchial basement membrane thickening and subepithelial fibrosis caused by fibronectin and collagen deposition, goblet cell metaplasia and mucus overproduction, smooth muscle hyperplasia and angiogenesis [1, 4], which eventually lead to irreversible airway stenosis, airflow obstruction and loss of lung function. Therefore, therapeutic interventions aimed at airway remodeling are urgently needed to prevent long-term impairment of lung function and improve the quality of life of asthmatic patients.

Circulating fibrocytes, a population of bone marrow-derived mesenchymal progenitors with fibroblastic properties [6], have been reported to be associated with tissue repair and airway remodeling. These fibrocytes can be isolated from peripheral blood mononuclear cells (PBMCs) and identified by co-expression of fibroblastic phenotype collagen I (COL-I) and leukocyte common antigen CD45 and/or hemopoietic stem cell marker CD34 [7]. Circulating fibrocytes not only secrete a variety of cytokines that are closely related to angiogenesis, inflammation, and fibrosis, but also can be recruited to bronchial tissues and differentiate into fibroblasts [7], which are involved in extracellular matrix (ECM) remodeling and tissue fibrosis. The accumulation of circulating fibrocytes in the lung was directly linked with the severity of airflow obstruction and bronchial subepithelial fibrosis in asthmatic patients [6]. Activation and migration of circulating fibrocytes are closely related to transforming growth factor-β1 (TGF-β1) and chemokine receptor CXCR4/CXCL12 signaling pathway [6]. However, how to regulate the recruitment of circulating fibrocytes to the airway and their subsequent differentiation and pro-fibrotic function to inhibit airway remodeling in asthma remains to be fully explored.

CD147, alternatively referred to as EMMPRIN or Basigin [8], is a transmembrane glycoprotein of the immunoglobulin superfamily, and its upregulation is related to the pathogenesis of asthma and lung inflammatory disease [9]. CD147 has been reported to be involved in the regulation of fibroblast activation and ECM homeostasis [10], as well as cell differentiation induced by TGF-β1 [11], and activation of the CXCR4/CXCL12 signaling pathway [12]. Treatment with anti-CD147 monoclonal antibody (mAb) can reduce lung inflammation and airway mucus secretion in asthmatic mice [13]. However, whether CD147 affects airway remodeling and the activation of circulating fibrocytes in asthma remains to be investigated.

In this study, a mouse model of asthma was established by sensitizing and challenging mice with ovalbumin (OVA). Mice were then treated with anti-CD147 mAb to investigate its role in airway remodeling and circulating fibrocytes. As a result, treatment of anti-CD147 repressed OVA-induced airway inflammation and airway remodeling in asthmatic mice, along with inhibiting the accumulation of circulating fibrocytes and their profibrotic function. Thus, CD147 may be the promising target for airway remodeling and the activation of circulating fibrocytes in asthma.

## Materials and methods

### Animal and ethical statements

Female BALB/c mice (Shanghai Slack Laboratory Animal Co., China), 6 weeks old, were housed in a specific pathogen-free environment and fed ad libitum. Animal experiments were approved by the Ethics Committee for the management and welfare of laboratory animals of the Northwest University, Xi’an, China. Animal experimental protocols were carried out in line with the Guidelines for the Care and Use of Laboratory Animals.

### Mouse model of asthma

Asthmatic mouse model was established with ovalbumin (OVA) and alum as previously described [14]. BALB/c mice were randomly divided into control group, OVA group, OVA + anti-CD147 group, and OVA + Isotype group, with 9 mice in each group. Mice in OVA group were intraperitoneally injected with 200 µL of sensitization solution containing 20 µg of OVA (Sigma, St. Louis, MO, USA) mixed with 500 µg of Al (OH)_3_ suspended in phosphate buffer saline (PBS), at Day 0, Day 7, and Day 14. From Day 21 to Day 61, the mice were challenged with 5% aerosolized OVA/PBS solution every other day by a nebulized inhalation system for 30 min per day. The mice in OVA + anti-CD147 group and OVA + Isotype group were intraperitoneally injected with anti-CD147 mAb (Abcam, Cambridge, UK; ab188190) or isotype IgG (1 µg per g of body weight) at 1 h before each OVA challenge. The control group was sensitized and challenged with PBS. All mice were sacrificed at 24 h after the last challenge (supplementary Fig. 1).

### Collection and testing of bronchoalveolar lavage fluid

The bronchoalveolar lavage fluid (BALF) samples were immediately collected after mice were sacrificed, as previously described [2]. Briefly, the mice tracheas were cannulated and lavaged three times with 0.5 mL of cold PBS. BALF samples were then centrifuged at 4 °C, the supernatants were collected stored at − 80 °C until use. Cell pellets were resuspended with PBS and used for smear analysis with Wright-Giemsa stain (Solarbio, Beijing, China), the number of total cells and eosinophils was counted by hemocytometer under a microscope [14]. The levels of CD147, interleukin-4 (IL-4), IL-5 and IL-13 in BALF supernatants were measured by enzyme-linked immunosorbent assay (ELISA) kits (Sino Biological Inc., Beijing, China).

### Histopathology of lungs

Lung tissues of experimental mice were fixed with 4% paraformaldehyde and prepared into paraffin-embedded tissue Sect. (4 μm). Sections were stained with hematoxylin and eosin (HE; Solarbio) to observe morphology changes, basement membrane thickness, and inflammatory cell infiltration [15]. Airway mucus secretion and goblet cell hyperplasia were identified by periodic acid-Schiff (PAS; Solarbio) staining and evaluated by PAS score based on the percentage of the areas of goblet cells in the airways: grade 0 (≤ 5%), grade 1 (5 − 25% ), grade 2 (25 − 50% ), grade 3 (50 − 75%), grade 4 (≥ 75%) [15]. Masson trichrome staining (Baso, Wuhan, China) was performed to assess airway collagen deposition and subepithelial fibrosis [15]. Five fields containing bronchial cross-sections were randomly selected for each staining, observed under a light microscope (Nikon, Tokyo, Japan), and images were analyzed by Image J software.

### Immunohistochemistry

The expression of CD31 was measured by immunohistochemistry to evaluate the microvascular density. After blocked with 5% normal goat serum for 10 min, tissue sections were incubated at 37 °C with a rabbit anti-mouse CD31 antibody (Abcam; ab182981; 1:2000 dilution) for 2 h, and then reacted with horseradish peroxidase-linked goat-anti-rabbit IgG (Abcam; ab6721; 1:1000 dilution) for 30 min. Finally, the sections were visualized with diaminobenzidine tetrahydrochloride (DAB; Sigma), and the intensity of the pale yellow or yellow-brown color in the peribronchial area was analyzed with Image J software.

### Immunohistofluorescence

Immunohistofluorescence staining was used to evaluate airway remodeling and identify the circulating fibrocytes. After blocked with 5% normal goat serum, tissue sections were incubated at 4 °C with rabbit anti-mouse α-Smooth muscle actin (α-SMA; Abcam; ab12964, 1:500 dilution), rat anti-mouse CD45 (Abcam; ab23910, 1:500 dilution) and rabbit anti-mouse collagen-I (COL-I; Abcam; ab270993, 1:2000 dilution) antibodies overnight. After reacted with FITC-labelled goat anti-rabbit IgG (Abcam; ab150077, 1:500 dilution) and Alexa Fluor 594-labelled goat anti-rat IgG (Abcam; ab150160, 1:500 dilution) at room temperature for 1 h, cell nuclei were stained with DAPI. Images was taken under Fluorescence microscope and analyzed by Image J software.

### Western blot assay

After homogenized and lysed as previously described [14], total proteins from lung tissues were extracted by centrifugation and quantitated with BCA Protein Assay Kit (Beyotime, Shanghai, China). Protein samples containing 20 µg of protein per lane were separated by SDS-PAGE and electroblotted to PVDF membranes (Millipore, Bedford, MA, USA). Membranes were then blocked with 5% nonfat milk, and incubated overnight at 4 °C with primary antibodies at specific dilutions (1:10,000 for GAPDH and α-SMA, 1:5000 for VEGF, 1:1000 for COL-I, Fibronectin and TGF-β1, all antibodies obtained from Abcam). The protein bands were visualized by an enhanced chemiluminescence system (Beijing 4 A Biotech Co., Ltd., Beijing, China), after 1 h of incubation with the corresponding secondary antibodies at room temperature. The intensity of the immunoreactive bands was analyzed with Image J software.

### Flow cytometry analysis of circulating fibrocytes

The percentage of CD45 and COL-I double-positive cells was measured by Flow cytometry to assess the number of circulating fibrocytes. In short, cell pellets obtained from BALF by centrifugation were re-suspended in 100 µL of PBS, and incubated with PE-conjugated CD45 antibody (BD Biosciences, San Jose, CA, USA) for 15 min at 4 °C. Then, cells were treated with Fix/Perm buffer, and reacted with FITC-linked COL-I antibody (BD Biosciences) for 30 min in the dark. Percentage of fibrocytes was analyzed with flow cytometer.

### Statistical analysis

All experimental data are presented as mean ± standard deviation (SD) of at least three replicates. Statistical analysis and data visualization were performed using SPSS 22.0 and GraphPad Prism 8.0 software. One-way analysis of variance (ANOVA) followed by Bonferroni correction was used to compare differences between multiple groups. The significant difference was identified with P values less than 0.05 (P < 0.05).

## Results

### Neutralization of CD147 reduces OVA-induced airway inflammation and histological changes in asthmatic mice

**Fig. 1:**
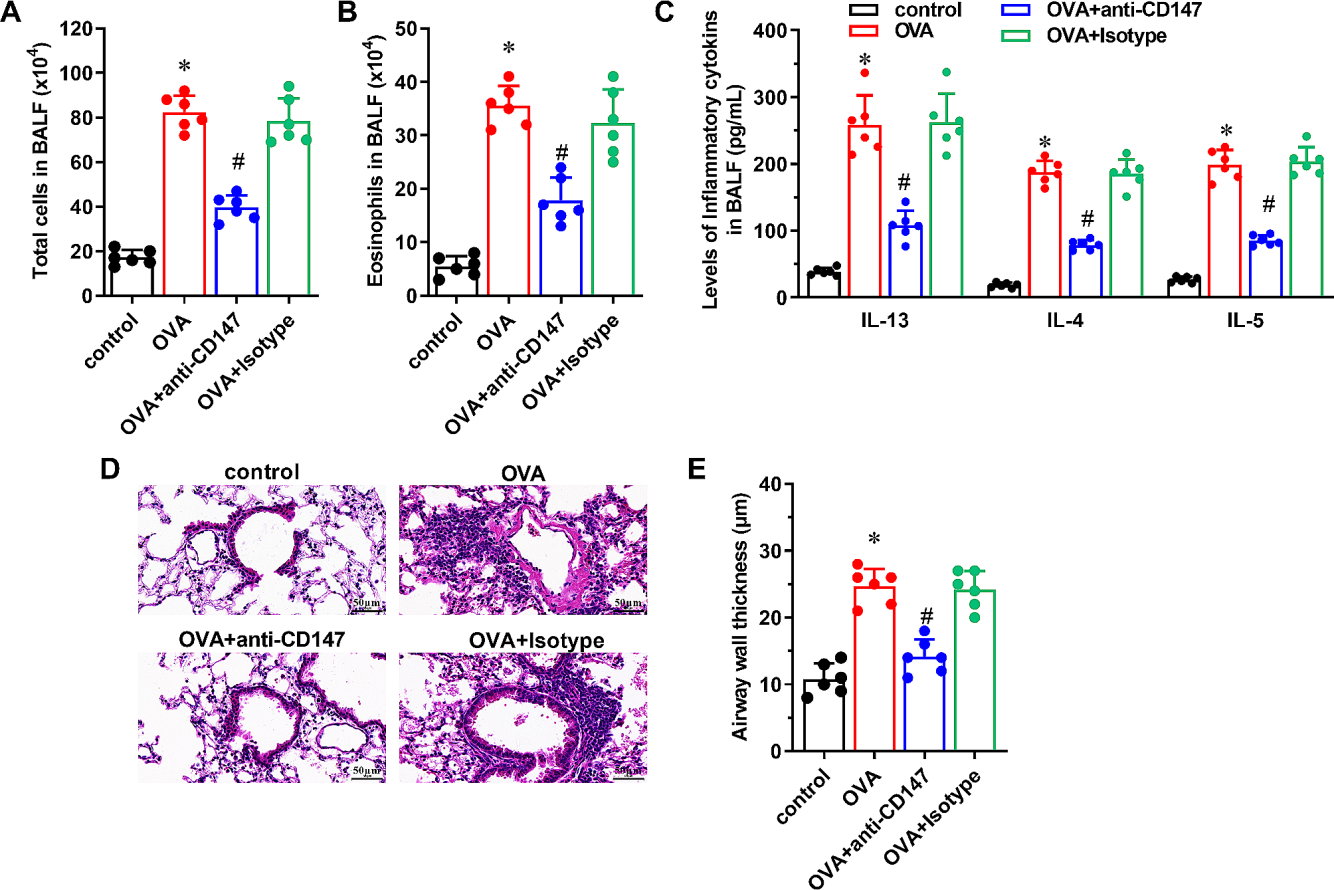
The effect of anti-CD147 in OVA-induced airway inflammation and histological changes of asthmatic mice Asthmatic mouse model was established with OVA, and treated with anti-CD147 or Isotype antibody. Mice sensitized and challenged with PBS were used as the control. The number of (**A**) total cells and (**B**) eosinophils in BALF of mice in each group were counted under a microscope (n = 6). (**C**) The levels of IL-4, IL-13, and IL-5 in BALF were assayed by ELISA (n = 6). (**D**) Representative photographs of lung sections were stained with HE, the nuclei appear blue, the cytoplasm appears pink. Magnification ×400, scale bar = 50 μm. (**E**) Quantification of airway wall thickness of mice in each group (n = 6). Data were presented as mean ± SD, and analyzed by one-way ANOVA with Bonferroni correction. *P < 0.05, vs. control; ^#^*P* < 0.05, vs. OVA

Compared with the control group, the mice in OVA group had significantly increased numbers of total cells and eosinophils in BALF (Fig. 1A and B, P < 0.05), and increased levels of CD147 (supplementary Fig. 2). Increased IL-4, IL-13 and IL-5 levels were observed in BALF of OVA-challenged mice as compared to the control group (Fig. 1C, P < 0.05). HE staining of lung tissues displayed increased inflammatory infiltration and airway stenosis in OVA-challenged mice as compared to the control group (Fig. 1D, P < 0.05). The airway wall thickness was also increased in OVA-challenged mice than that in the control group (Fig. 1E, P < 0.05). However, the number of total cells and eosinophils and the levels of IL-4, IL-13, and IL-5 in BALF were significantly reduced in OVA + anti-CD147 group, as compared to the OVA group (Fig. 1A and C, P < 0.05). The inflammatory infiltration, airway stenosis and airway wall thickness were also significantly reduced in OVA + anti-CD147 group when compared with the OVA group (Fig. 1D and E, P < 0.05).

### Neutralization of CD147 reduces OVA-induced airway goblet cell metaplasia and collagen deposition in asthmatic mice

**Fig. 2:**
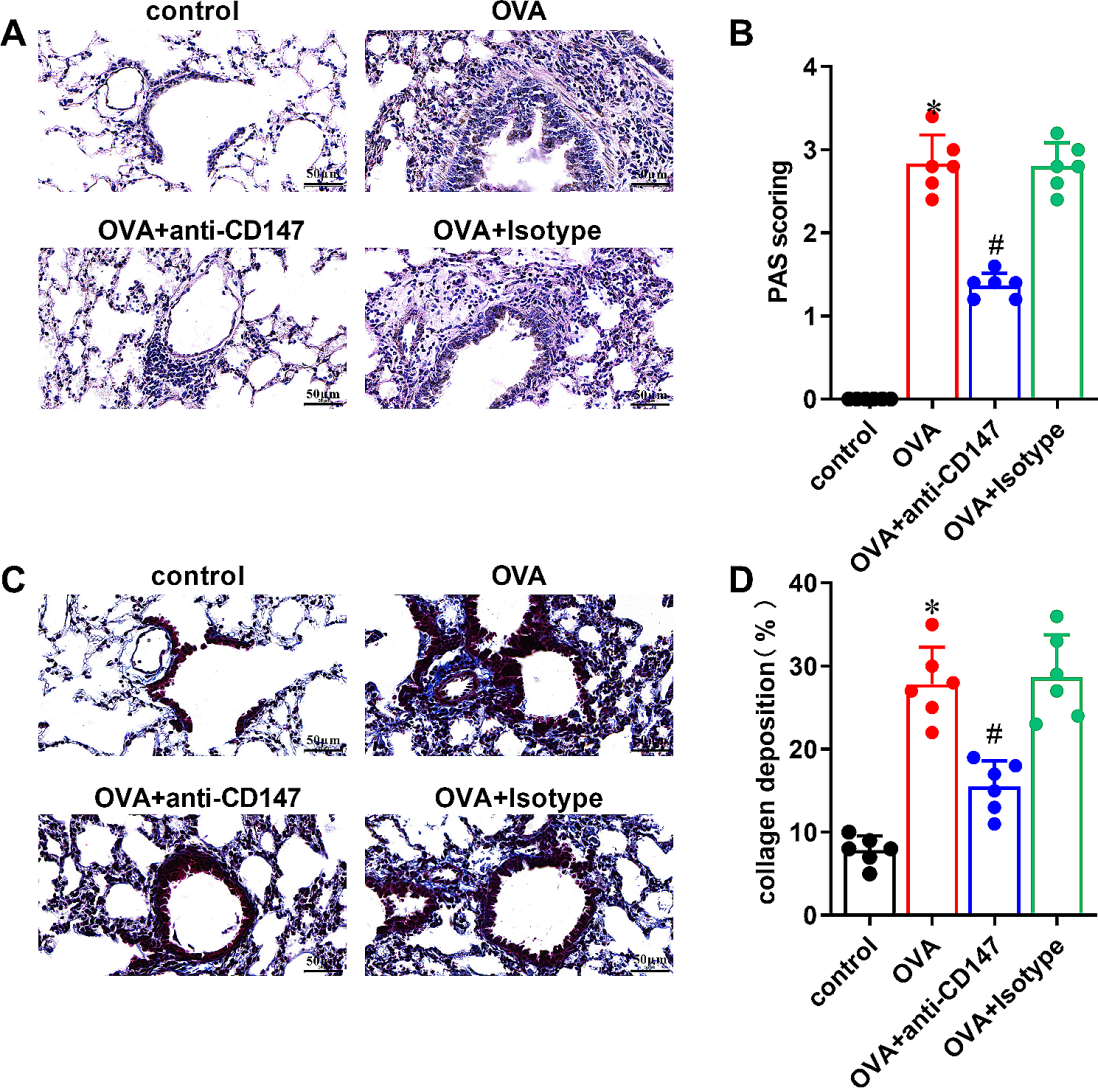
The effect of anti-CD147 in OVA-induced airway goblet cell metaplasia and collagen deposition in asthmatic mice OVA-induced asthmatic mice were treated with anti-CD147 or Isotype antibody. Mice treated only with PBS were considered as the control. (**A**) Representative photographs of lung sections stained with PAS; bronchial goblet cells appear fuchsia. Magnification ×400, scale bar = 50 μm. (**B**) Scoring of PAS based on PAS staining (n = 6). (**C**) Representative photographs of lung sections stained with Masson trichrome, collagen fibers appear blue, muscle fibers and cytoplasm appear red, and the nuclei appear blue-black. Magnification ×400, scale bar = 50 μm. (**D**) Quantification of peribronchial collagen deposition based on Masson staining (n = 6). Data were presented as mean ± SD, and analyzed by one-way ANOVA with Bonferroni correction. *P < 0.05, vs. control; ^#^*P* < 0.05, vs. OVA

Compared with the control group, PAS staining of lung tissues showed increased airway goblet cell metaplasia in OVA group (Fig. 2A and B, P < 0.05). Masson trichrome staining displayed increased collagen deposition in OVA group, as compared to the control group (Fig. 2C and D, P < 0.05). However, compared with the OVA group, airway goblet cell metaplasia and collagen deposition were significantly decreased in OVA + anti-CD147 group (Fig. 2A and D, P < 0.05)

### Neutralization of CD147 reduces OVA-induced vascular remodeling in asthmatic mice

**Fig. 3:**
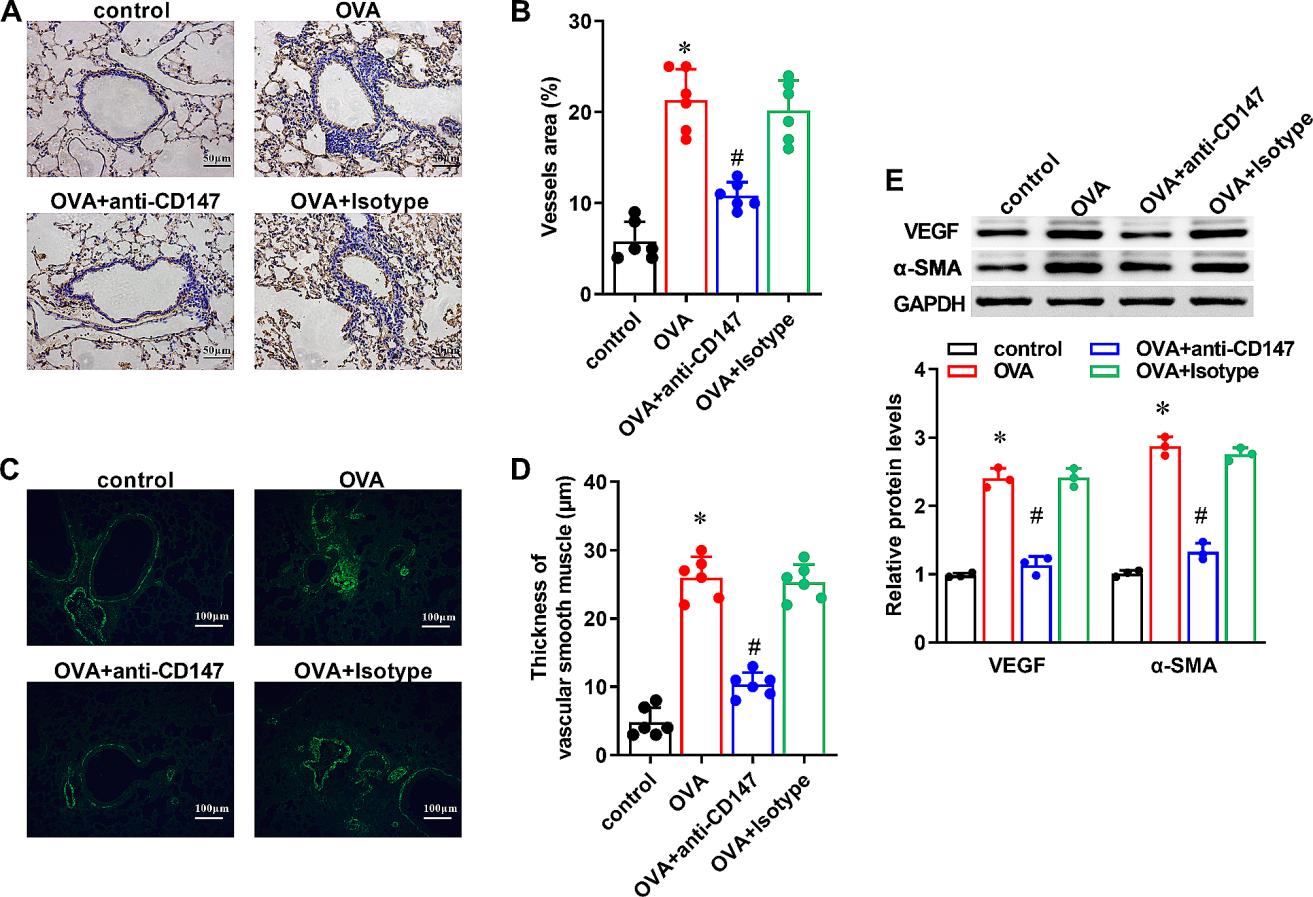
The effect of anti-CD147 in OVA-induced vascular remodeling in asthmatic mice Mice asthma models were induced by OVA, and treated with anti-CD147 or Isotype antibody. Mice treated only with PBS were used as controls. (**A**) Representative immunohistochemical images of CD31 protein in lung sections, its positive signal appears pale yellow or yellow-brown and nuclei appear blue. Magnification ×400, scale bar = 50 μm. (**B**) Quantification of vessels area (n = 6). (**C**) Representative immunohistofluorescence images of α-SMA protein in lung sections, its positive signal appears green. Magnification ×200, scale bar = 100 μm. (**D**) Quantification of the thickness of vascular smooth muscle around the bronchioles (n = 6). (**E**) Representative western blotting images of VEGF, α-SMA and quantification of their protein expression normalized to GAPDH (n = 3). Data were presented as mean ± SD, and analyzed by one-way ANOVA with Bonferroni correction. *P < 0.05, vs. control; ^#^*P* < 0.05, vs. OVA

CD31 staining of lung tissues showed increased CD31 expression and increased area of vessels in OVA group when compared to the control group (Fig. 3A and B, P < 0.05). The thickness of vascular smooth muscle around the bronchioles was also increased in OVA group than that in the control group, as evaluated by immunohistofluorescence of α-SMA (Fig. 3C and D, P < 0.05). However, compared with the OVA group, angiogenesis and smooth muscle thickness were significantly decreased in the OVA + anti-CD147 group (Fig. 3A and D, P < 0.05). Moreover, the protein expression of VEGF and α-SMA was increased in the OVA group than that in the control group, but decreased in the OVA + anti-CD147 group when compared to the OVA group (Fig. 3E, P < 0.05)

### Neutralization of CD147 reduces the recruitment of circulating fibrocytes in the airway of asthmatic mice

**Fig. 4:**
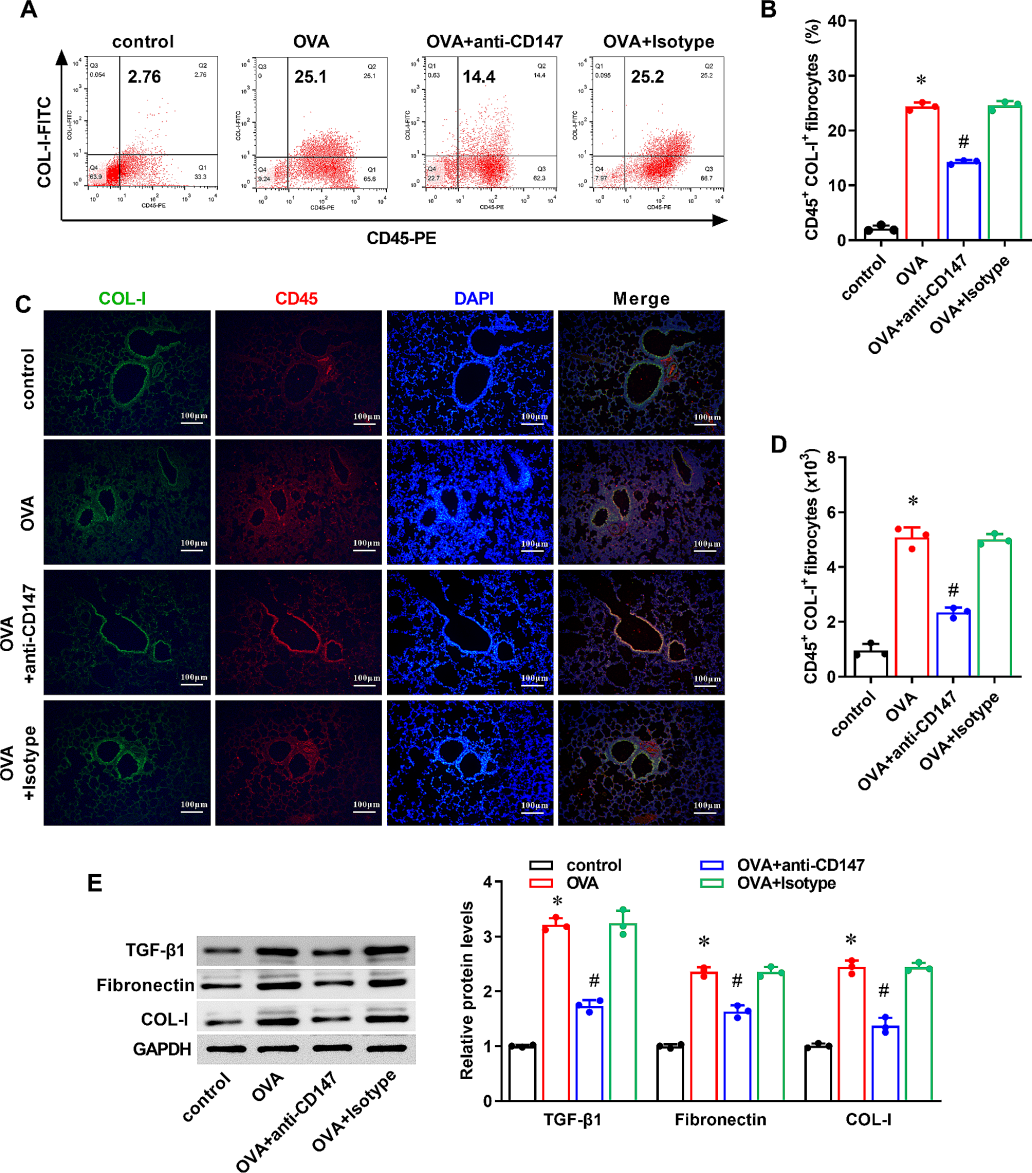
The effect of anti-CD147 in OVA-induced recruitment of circulating fibroblasts in asthmatic mice Asthmatic mice were induced by OVA, and treated with anti-CD147 or Isotype antibody. Mice treated only with PBS were used as the control. (**A**) Representative flow cytometry images revealed the percentage of CD45^+^ and COL-I^+^ cells. (**B**) Quantification of CD45^+^COL-I^+^ fibrocytes in BALF of mice (n = 3). (**C**) Representative immunohistofluorescence images stained with antibodies to CD45 and COL-I revealed the presence of CD45^+^COL-I^+^ fibrocytes in lung tissue sections. COL-I appears green, CD45 appears red, and nuclei appear blue. Magnification ×200, scale bar = 100 μm. (**D**) Quantification of the number of CD45^+^COL-I^+^ fibrocytes in lung tissue sections (n = 3). (**E**) Representative western blotting images of COL-I, Fibronectin and TGF-β1 and quantification of their protein expression normalized to β-Tubulin (n = 3). Data were presented as mean ± SD, and analyzed by one-way ANOVA with Bonferroni correction. *P < 0.05, vs. control; ^#^*P* < 0.05, vs. OVA

According the analysis of flow cytometry, CD45 and COL-I double positive cells were increased in BALF of asthmatic mice in the OVA group compared to the control group (Fig. 4A and B, P < 0.05). However, compared with the OVA group, the number of CD45^+^COL-I^+^ cells were significantly decreased in the OVA + anti-CD147 group (Fig. 4A and B, P < 0.05). Similarly, the recruitment of circulating fibrocytes in lung tissues was also increased in the OVA group than that in the control group (Fig. 4C and D, P < 0.05), as evaluated by immunohistofluorescence of CD45 and COL-I. The amount of CD45^+^COL-I^+^ cells was significantly decreased in OVA + anti-CD147 group compared to the OVA group (Fig. 4C and D, P < 0.05). Moreover, compared with the control group, the protein expression of COL-I, Fibronectin and TGF-β1 in lung tissues of the mice was also increased in the OVA group (Fig. 4E, P < 0.05). And, compared with the OVA group, the protein expression of COL-I, Fibronectin and TGF-β1 were all downregulated in the OVA + anti-CD147 group (Fig. 4E, P < 0.05).

## Discussion

As a critical low-reversibility features of asthma, airway remodeling is strongly associated with increased morbidity and mortality in patients with asthma. However, current medications for asthma have only little effect on airway remodeling [16]. CD147 is a class of multipotent molecules that can participate in matrix metalloproteinases (MMPs) synthesis and ECM lysis, cell differentiation and migration, cell-matrix interactions, angiogenesis, etc. [17]. Although CD147 is closely related to the pathogenesis of asthma and pulmonary inflammatory diseases [9], its roles in airway remodeling have been largely unknown. In this study, OVA-induced asthmatic mice were treated with anti-CD147 mAb. As a result, anti-CD147 treatment inhibited airway inflammation and airway remodeling in OVA-induced asthmatic mice. And, the mechanism may be related to the inhibitory effect of anti-CD147 treatment on the accumulation of circulating fibrocytes and their profibrotic function.

Airway inflammation is one of the typical pathological features of asthmatic individuals, accompanied by an increase in eosinophils and Th2-associated pro-inflammatory cytokines IL-4, IL-13, and IL-5 [18]. As expected, asthmatic mice were successfully established with OVA, along with the increased amounts of eosinophils, IL-4, IL-13, and IL-5 in BALF. Moreover, HE staining of lung tissue showed significant histological changes, including inflammatory infiltration, airway wall thickening, and airway stenosis, which are also typical of asthmatic mice [19]. Similar to previous findings [13], anti-CD147 treatment reduced the number of eosinophils in BALF, and repressed the production of IL-4, IL-13, and IL-5 in BALF. As the pivotal factors contributing to airway remodeling [20], IL-4, IL-13, and IL-5 reportedly can induce the production of TGF-β1 and α-SMA, increase collagen deposition and goblet cell metaplasia, and promote subepithelial and peribronchial fibrosis [21].

As an extracellular matrix metalloproteinase inducer, CD147 has been reported to participate in tissue remodeling by regulating ECM deposition and degradation, cell adhesion, cell-cell interactions, and inducing myofibroblast differentiation [8]. Moreover, CD147 can modulate pulmonary fibrosis [22], liver fibrosis [23], cardiac fibrosis [24], along with regulation of MMPs, TGF-β1, α-SMA, or VEGF expression. Thus, whether and how anti-CD147 affect airway remodeling were investigated, and the result revealed that anti-CD147 treatment reduced airway goblet cell metaplasia, collagen deposition, and vascular remodeling in OVA-induced asthmatic mice, accompanied by inhibition of VEGF and α-SMA expression. These findings are similar to previous studies reported that CD147 can modulate airway mucus secretion [25], and promote myofibroblast differentiation by regulating collagen gel contraction and α-SMA expression [26]. In addition, angiogenesis is one of the important processes of airway remodeling [27], and CD147 reportedly plays a crucial role in the process of angiogenesis [17].

As one of the biological markers to evaluate the progression of chronic lung-related diseases, circulating fibrocytes have been shown to influence airway repair and remodeling in bronchial asthma through the expression of pro-inflammatory and pro-fibrotic cytokines, differentiation into (muscle) fibroblasts, and ECM remodeling [6]. Studies have shown that the number of circulating fibrocytes increases in peripheral blood of patients with bronchial asthma, and it is closely related to the restricted airflow in the airway of asthmatic patients [28]. In addition, the number, activation, and differentiation of circulating fibrocytes are correlated with the severity of asthma [29]. Inhibition of the chemotaxis, proliferation, and fibrous transformation of circulating fibrocytes can help reduce airway basement membrane thickening [7]. TGF-β1 and CXCR4/CXCL12 signaling pathway have been shown to affect proliferation, differentiation, and chemotaxis of circulating fibrocytes [6], while CD147 has been reported to affect TGF-β1-mediated cell differentiation and CXCR4/CXCL12 signal transduction pathway [11, 12]. However, whether CD147 affects the recruitment and activation of circulating fibrocytes are largely unclear. In the present study, anti-CD147 treatment significantly decreased the number of circulating fibrocytes, which are regarded as CD45^+^COL-I^+^ cells, in both BALF and lung tissues of asthmatic mice. In addition, anti-CD147 treatment also reduced the protein expression of COL-I, fibronectin, and TGF-β1 in lung tissues of asthmatic mice. These findings are consistent with the previous study reported that CD147 promotes fibrosis in a variety of tissues and regulates the expression of fibrosis-related proteins such as TGF-β1 [30, 31]. However, these findings still need to be further validated and supported by more experimental techniques and clinical data, which is one of the limitations of this study.

## Conclusion

In summary, anti-CD147 treatment can alleviate OVA-triggered airway inflammatory injury and airway remodeling in asthmatic mice, and repress the accumulation of circulating fibrocytes and their profibrotic function. CD147 may be the promising target for airway remodeling and the activation of circulating fibrocytes in asthma.

### Electronic supplementary material

Below is the link to the electronic supplementary material.

Supplementary Material 1

## Data Availability

The datasets used and/or analysed during the current study are available from the corresponding author on reasonable request.

## Abbreviations

OVA: ovalbumin
BALF: bronchoalveolar lavage fluid
IL-4: interleukin-4
COL-I: collagen I
HE: hematoxylin and eosin
PAS: periodic acid-Schiff
α-SMA: α-Smooth muscle actin
TGF-β1: transforming growth factor-β1
ELISA: enzyme-linked immunosorbent assay
ECM: extracellular matrix
PBS: phosphate buffer saline
MMPs: matrix metalloproteinases
VEGF: vascular endothelial growth factor
