# Supplementary material for: CD147 induces asthmatic airway remodeling and activation of circulating fibrocytes in a mouse model of asthma
Source: Respir Res. 2024 Jan 4;25:6. doi: 10.1186/s12931-023-02646-5 (PMC10765784; doi:10.1186/s12931-023-02646-5)
Supplement: Supplementary file 1 — Supplementary Material 1 [file 12931_2023_2646_MOESM1_ESM.docx]

**Supplementary materials**

**Figure S1**


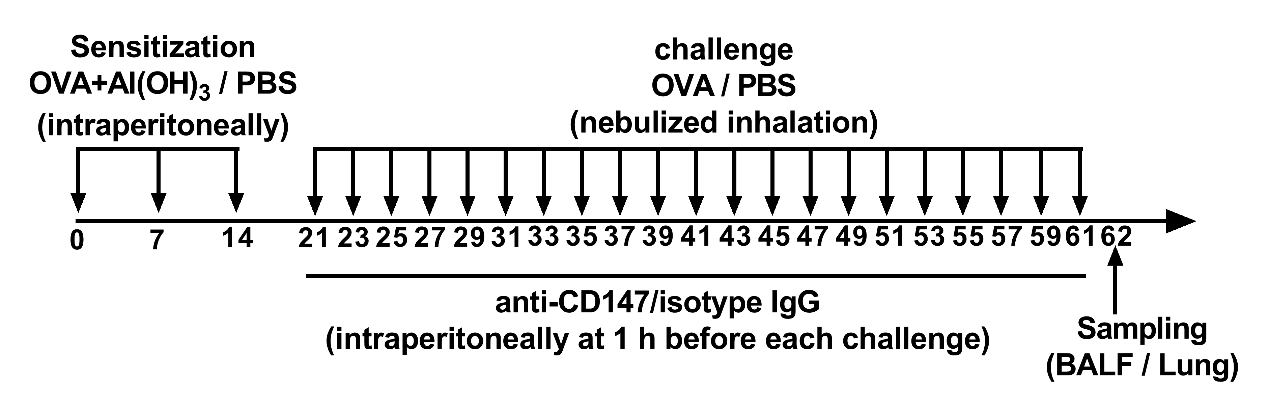


**Figure S1 Schematic illustration of the establishment of** **airway remodeling model in asthmatic mice.** BALB/c mice intraperitoneally sensitized by OVA mixed with Al (OH)_3_， or PBS at Day 0, Day 7, and Day 14. From Day 21 to Day 61, the mice were challenged with OVA or PBS every other day by a nebulized inhalation system for 30 min per day. Anti-CD147 mAb or isotype IgG were intraperitoneally injected at 1 h before each OVA challenge. At Day 62, BALF, serum and lung tissue samples were collected.

**Figure S2**


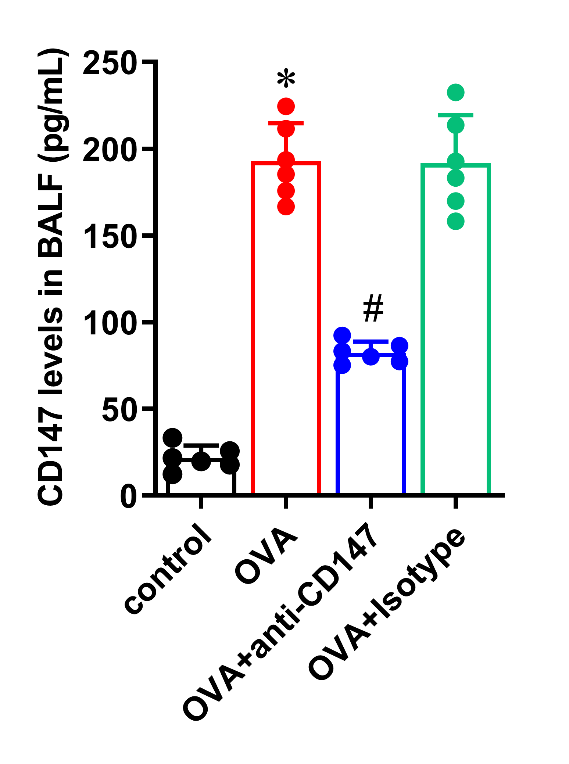


**Figure S2** **The level of CD147 in BALF of asthmatic mice.** Asthmatic mouse model was established with OVA, and treated with anti-CD147 or Isotype antibody. Mice sensitized and challenged with PBS were used as the control. The level of CD147 in BALF of mice was assayed by ELISA kit. Data represent means ± SD, n=6 for each group.
